# IgA-Dominant Infection-Associated Glomerulonephritis Following SARS-CoV-2 Infection

**DOI:** 10.3390/v13040587

**Published:** 2021-03-31

**Authors:** Aurora Pérez, Isidro Torregrosa, Luis D’Marco, Isabel Juan, Liria Terradez, Miguel Ángel Solís, Francesc Moncho, Carmen Carda-Batalla, María J. Forner, Jose Luis Gorriz

**Affiliations:** 1 Nephrology Department, Hospital Clínico Universitario, INCLIVA, Universidad de Valencia, 46010 Valencia, Spain; aurora91991@gmail.com (A.P.); isist67@gmail.com (I.T.); luisgerardodg@hotmail.com (L.D.); ijuangar@hotmail.com (I.J.); miguelsolis7@yahoo.es (M.Á.S.); FrancescMoncho@gmail.com (F.M.); 2 Pathology Department, Hospital Clínico Universitario, INCLIVA, Universidad de Valencia, 46010 Valencia, Spain; liria.terradez@gmail.com (L.T.); carmen.carda@uv.es (C.C.-B.); 3 Internal Medicine Department, Hospital Clínico Universitario, INCLIVA, Universidad de Valencia, 46010 Valencia, Spain; Maria.Jose.Forner@uv.es

**Keywords:** SARS-CoV-2, glomerulonephritis, acute kidney injury, COVID-19

## Abstract

The renal involvement of severe acute respiratory syndrome coronavirus 2 (SARS-CoV-2) has been reported. The etiology of kidney injury appears to be tubular, mainly due to the expression of angiotensin-converting enzyme 2, the key joint receptor for SARS-CoV-2; however, cases with glomerular implication have also been documented. The multifactorial origin of this renal involvement could include virus-mediated injury, cytokine storm, angiotensin II pathway activation, complement dysregulation, hyper-coagulation, and microangiopathy. We present the renal histological findings from a patient who developed acute kidney injury and de novo nephrotic syndrome, highly suggestive of acute IgA-dominant infection-associated glomerulonephritis (IgA-DIAGN) after SARS-CoV-2 infection, as evidenced by the presence of this virus detected in the renal tissue of the patient via immunohistochemistry assay. In summary, we document the first case of IgA-DIAGN associated to SARS-CoV-2. Thus, SARS-CoV-2 S may act as a super antigen driving the development of multisystem inflammatory syndrome as well as cytokine storm in patients affected by COVID-19, reaching the glomerulus and leading to the development of this novel IgA-DIAGN.

## 1. Introduction

Beyond the expected pulmonary involvement in severe acute respiratory syndrome coronavirus 2 (SARS-CoV-2), impact on other organs has also been reported [1], and among them, kidney involvement of this novel virus has been documented in patients affected by coronavirus infection disease 2019 (COVID-19). The etiology of kidney damage appears to be tubular, mainly due to the expression of angiotensin-converting enzyme 2 (ACE2), the key joint receptor for SARS-CoV-2 [2]. However, some cases with glomerular implication have also been reported [3,4,5,6]. The multifactorial origin of this renal involvement could include virus-mediated injury, cytokine storm, angiotensin II pathway activation, complement dysregulation, hyper-coagulation, and microangiopathy [7,8].

The present report shows the renal histological findings from biopsy in a patient who developed acute kidney injury (AKI) and de novo nephrotic syndrome. Our findings are highly suggestive of acute IgA-dominant infection-associated glomerulonephritis (IgA-DIAGN) after SARS-CoV-2 infection, as demonstrated by presence of the virus in renal tissue with immunohistochemistry assay.

## 2. Case Presentation

We report the case of an 88-year-old male non-smoker or drinker with no known history of renal disease, whose medical record included essential hypertension, dyslipidemia, depression and essential tremor. He was on regular treatment with valsartan 160 mg/day, amlodipine 5 mg/day, propranolol 10/TID and atorvastatin 10 mg/day. The patient’s lab record is shown in Table 1.

After diagnosis with COVID-19 (PCR and serology positive for SARS-CoV-2), the patient was hospitalized for 33 days due to respiratory insufficiency. Baseline serum creatinine a month before COVID-19 diagnosis was 1 mg/dL, with an estimated glomerular filtration rate (eGFR) of 67 mL/min/1.73 m^2^ with normal urinalysis. During hospitalization, the patient was treated with hydroxychloroquine, lopinavir/ritonavir, β interferon (IFN), azithromycin and steroids as per current COVID-19 protocol at the time. At hospital admission, serum creatinine was 1.48 mg/dL; however, at the time of discharge, levels had decreased to 0.8 mg/dL. Urine lab stick tests reported proteinuria 70 mg/dL, red blood cells >100 and white blood cells 1–5. Urine culture was negative. During hospital follow-up, no other infection was documented. After recovery, the patient was discharged to home. Interestingly, the PCR for SARS-CoV-2 remained positive seven days later.

Three weeks after discharge, the patient was referred again to the hospital for dyspnea and edema. During admission evaluation, oxygen saturation was 87%. Physical examination showed peripheral edema and decreased respiratory sounds. Chest X ray reported a COVID-19 pattern with right pleural effusion. No other findings were detected. Abdominal ultrasound was normal. Laboratory analysis was notable for AKI (Table 1) with serum creatinine of 2.28 mg/dL; urinalysis showed >600 mg/dL proteinuria, >100 RBC/HPF (20–40% acanthocytes), 10–20 WBC/HPF and granular casts. Urinary osmolality was 1030 and urinary sodium 80 mmol/L. PCR (for SARS-CoV-2) at that moment was negative but serology (total antibodies, IgM and IgG) was positive.

Immediately after the second admission, IV furosemide was initiated. Proteinuria reached 3.76 g/day, with a urinary creatinine/protein ratio of 5.79 gr/gr, and serum albumin of 3 g/dL. The serologic panel screens were negative for HIV, C and B hepatitis. Circulating immune complex, anti-nuclear, anti-DNA, anti-cardiolipin, ANCA, anti-GBM, and cryoglobulin tests were also negative. Serum C3 complement was normal (97 mg/dL) while C4 was low (9 mg/dL). Rheumatoid factor was high (174 UI/mL, range 0–14). Serum protein electrophoresis and immunofixation showed monoclonal bands of IgG-kappa, IgM-kappa and serum lambda lights chain. Urinary RNA for SARS-CoV-2 was negative. A summary of the remaining laboratory analysis and significant trends during hospitalization are shown in Table 1.

During the first week in hospital, renal function worsened progressively, with serum creatinine levels of 3.3 mg/dL, and a course of corticoids was initiated (IV methylprednisolone 250 mg × 3 followed by PO prednisone 1 mg/kg/day). An echocardiogram ruled out endocarditis. Kidney biopsy was performed on Day 5 of hospitalization, one day after initiating steroid therapy.

## 3. Kidney Biopsy Findings

**Figure 1:**
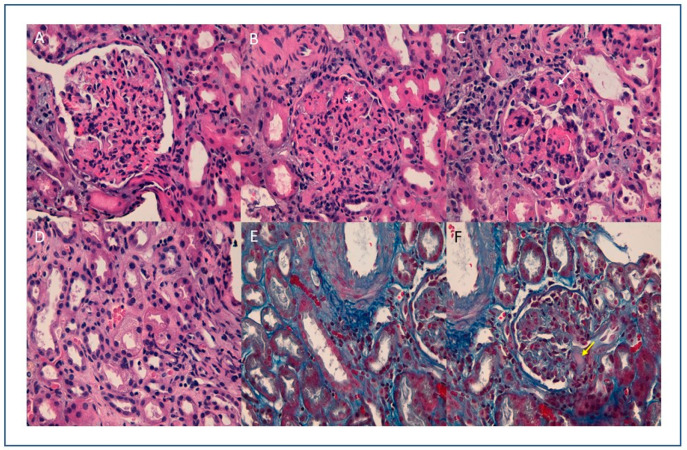
(**A**) (10×) (**B**) (20×) (**C**) (40×) H.E: Glomeruli with endocapillary proliferation, observing collapsed vascular lumens and increased cellularity with isolated neutrophils (white arrow) and nuclear fragments (*). Thrombi are not observed; (**D**) (20×), HE: Tubules with isometric cytoplasmic vacuolization, (**E**) (10×) (**F**) (40×) Trichrome stain: erythrocytes in peritubular capillaries and solitary glomerular hyaline thrombus (yellow arrow).

An ultrasound-guided percutaneous renal biopsy was performed, yielding three samples for histological, direct immunofluorescence (DIF) and ultrastructural study. Microscopic study showed renal tissue including eight glomeruli with a diffuse endocapillary proliferative pattern, with associated neutrophilic exudation and focal nuclear dust (Figure 1). Glomerular capillaries showed occlusion of microvascular lumens mainly by erythrocytes, and a solitary hyaline thrombus was present (Figure 1). No double contours, fibrin thrombus, or mesangiolysis or myxoid degeneration of capillary walls suggestive of thrombotic microangiopathy were observed. Immunohistochemical study with C5B9 and CD61 was negative. Tubules showed non-isometric vacuolization, and some hyperchromatic nuclei, as reparative changes (Figure 1). A sparse lymphocytic interstitial infiltrate with scant eosinophils, with no tubulitis, occupied less than 5% of the cortical area. The peritubular capillaries also showed erythrocyte intraluminal aggregation, without platelets or thrombi.

**Figure 2:**
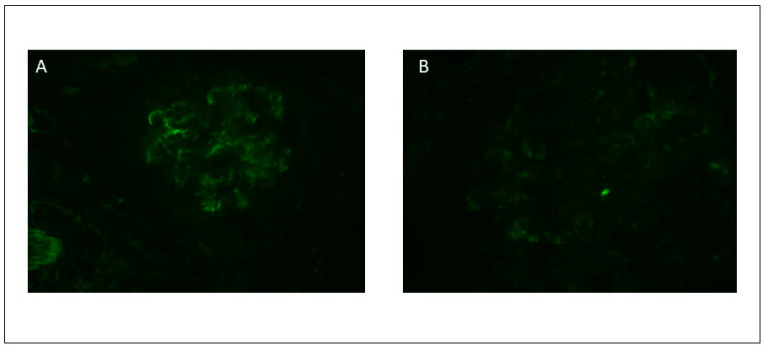
Direct immunofluorescence study showing predominant IgA positivity (**A**) and C3 positivity (**B**) with granular pattern and mesangial distribution.

On DIF we analyzed six glomeruli with a diffuse and intense IgA deposit with a granular pattern along the mesangial and glomerular capillary walls (Figure 2), and a segmental deposition of lower intensity for C3 and IgG. No light chain restriction was observed. Intense positivity with fibrinogen along the capillary walls was also observed, suggesting endothelial damage.

**Figure 3:**
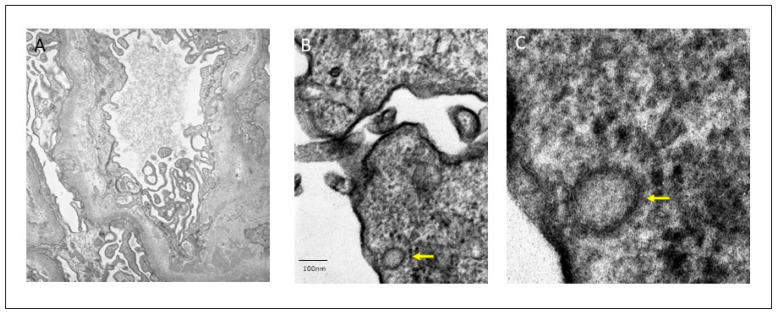
Electron-dense deposits with mesangial and para-mesangial location (**A**). Viral particles with a double contour membrane and a crown of 120 nm diameter in podocyte cytoplasm (arrows) (**B**,**C**).

**Figure 4:**
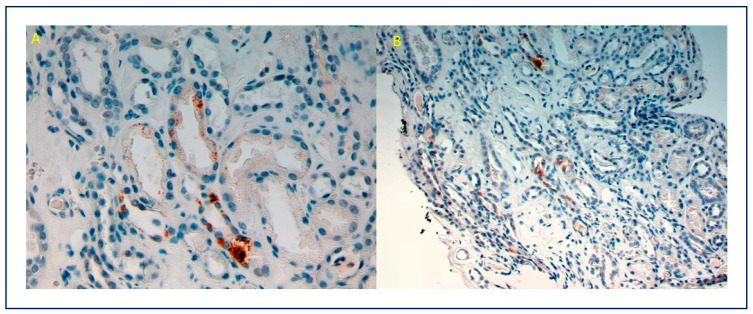
(**A**) (10×), (**B**) (20×): Positive immunostain for SARS-CoV-2 with a granular cytoplasmic pattern in tubular epithelial cells.

Ultrastructural electron-dense deposits were confirmed at the mesangial, para-mesangial, and focally at the sub-endothelial level. No sub-epithelial humps were found. Podocytes showed areas of foot process effacement and microvillus change. Endothelial swelling and electron-lucent thickening of subendothelial space were observed, without thrombi. Viral particles with double contour membrane and a characteristic corona, 120 nm sized, were localized in podocyte cytoplasm (Figure 3). Tubular epithelium showed isometric vacuoles. Immunohistochemical analysis with SARS-CoV-2 spike antibody (1A9) (monoclonal, Gene Tex) was performed in Autostainer 48 (pH low, dilution 1/200), showing granular cytoplasmic positivity in tubular epithelial cells, as described in the literature [9] (Figure 4). PCR for SARS-CoV-2 renal tissue was negative. These findings support the diagnosis of acute IgA-DIAGN after SARS-CoV-2 infection, with the presence of viral particles corroborated by immunohistochemical staining and ultrastructural study.

## 4. Follow-Up

During in-hospital follow-up, renal function slowly recovered after medical treatment, and improvement was observed regarding respiratory involvement. The patient was discharged after 12 days in hospital. Serum creatinine and proteinuria levels were 2.35 and 300 mg/dL, respectively (Table 1). Two months after discharge, serum creatinine value and proteinuria returned to basal ranges, with steroids being the only treatment given for glomerulonephritis (GN).

## 5. Discussion

To our knowledge, this is the first case of acute IgA-DIAGN after SARS-CoV-2 infection. On the basis of this discovery, we discuss the data and possible pathogenesis of IgA-DIAGN and renal damage associated with COVID-19.

IgA-DIAGN is a morphological variant in which, unlike classical post-infectious GN, which involves C3 and IgG or only C3 deposition, IgA is the sole or dominant immunoglobulin mediator [10]. In most acute post-infectious (AP) GN cases, the agent involved is staphylococcus aureus; however, there are some cases associated with infection by other germs such as *Streptococcus*, *Escherichia coli*, *Klebsiella pneumoniae*, *Chlamydia pneumoniae* and Hepatitis A virus, among others [10,11,12,13,14,15]. IgA-DIAGN typically appears in the elderly, especially in patients with multiple comorbidities [11,12,13,14].

As noted earlier, histologic findings are crucial for differentiating IgA-DIAGN from other cases of IgA GN or typical APGN. Notably, in several cases, diagnosis of infection was made at the time of renal biopsy, suggesting that the infection may go undetected for some time [10,12]. The most relevant clinical and analytical findings in IgA-DIAGN are AKI (84.6%), proteinuria (96.2%), and hematuria (97.4%). Complement is low in 57.3% of cases. On light microscopy (LM), the most common histologic pattern of glomerular damage is diffuse (both mesangial and endocapillary) proliferative GN, followed by endocapillary proliferative GN and mesangial proliferative GN. On immunofluorescence microscopy (IF) evaluation, IgA was the sole or dominant immunoglobulin deposited in glomeruli in all cases. There was also high-intensity staining for C3 in almost every reported case. On electronic microscopy (EM), mesangial, subepithelial and subendothelial electron-dense deposits were present in 87.3, 63.5, and 38.1% of reported cases, respectively. Subepithelial deposits frequently appeared as humps.

Various theories have been proposed regarding the pathogenesis of IgA-DIAGN, including that staphylococcus enterotoxin acts as a superantigen which can activate the immune system intensively, producing cytokines that activate B cells and produce polyclonal IgA and IgG, with deposit formation in the glomeruli [16,17,18].

Until now, no other cases of IgA-DIAGN related to SARS-Cov-2 have been reported. Our case presents clinical and histological characteristics very similar to those previously described in IgA-DIAGN by other agents; however, using immunostaining for SARS-CoV-2 demonstrates a high likelihood that this is the causal agent of the glomerular lesion.

In our case, the histological features of a diffuse endocapillary proliferative pattern, and DIF findings (with capillary wall deposits for IgA, IgG and C3) suggest an IgA-DIAGN with endocapillary proliferation and IgA-dominant deposits, although the typical humps at EM were not present. However, as we mentioned before the presence of humps is not pivotal to reach this diagnosis. In addition to this histological pattern, the capillary wall deposits on DIF, with intense positivity for fibrinogen along the capillary wall, were also suggestive of endothelial damage.

We postulated that these histological findings were compatible with GN, secondary to SARS-CoV-2 itself or to another superimposed infection, yet no other infection was documented in this patient. The possibility that the causative agent was prior treatment with IFN was also initially raised, in which context, this drug has been associated with a broad spectrum of glomerulopathies [19,20]. Nevertheless, the IgA-DIAGN emerged approximately seven weeks after IFN treatment, and the histological findings in this patient are unlikely to be related to interferon. Alternatively, presence of oligo clonal bands in serum and urine could have led to monoclonal gammopathy with renal significance, but this was not confirmed in the biopsy. We also evaluated the possibility of an asymptomatic IgA-GN exacerbated after SARS-CoV-2 or other infections. Nonetheless, we found no evidence of active urinary sediment nor others evidences of interest in the medical record (from 2008 to date). Finally, the causative agent infection could be attributed to SARS-CoV-2 due to positive immunostaining for SARS-Cov-2 in renal tissue.

A high prevalence of AKI, hematuria and proteinuria has been observed in studies of COVID-19 and renal involvement [8,21,22,23,24,25]; AKI has been reported in 36.6% of patients [26,27,28,29]. COVID-19-associated de novo glomerulopathy has also been described, and evidence of renal involvement in COVID-19 patients has been accumulating rapidly since the beginning of the pandemic. In the first three cases published, the morphological pattern is collapsing glomerulopathy [3,4,5,30].

Su et al. analyzed kidney abnormalities in 26 autopsies of COVID-19 patients [31]. The main histological finding by LM was diffuse proximal tubule involvement. EM examination showed clusters of coronavirus particles with distinctive spikes in the tubular epithelium and podocytes. Furthermore, immunostaining with SARS-CoV-2 nucleoprotein antibody was positive in tubules. This study therefore provided direct evidence of invasion of the virus into kidney tissue. Nonetheless, the presence of SARS-CoV-2 particles in EM and their meaning is controversial. Roufosse et al. raised doubts about the true meaning of ME findings in these published cases [32]. They showed images from three live COVID-19 patient biopsies from different centers, in which they found similar images to those reported in the literature. They concluded that the intracellular structures represented clathrin-coated (CC) vesicles and micro-vesicular bodies (MVB), whereas the extracellular structures represented extruded micro vesicles from MVB and degenerate microvilli. MVB may fuse with lysosomes and pits; their clathrin coat resembles a crown on EM. In this regard, Goldsmith et al. also express doubts about the correct interpretation of particles described as coronaviruses in other publications [33]. In our patient, we found a small number of particles in the renal tissue; thus, the morphology, size and location coincide with those described in the literature, and immunohistochemical analysis showed cytoplasmic positivity for SARS-CoV-2. Therefore, the localization of the virus in the renal tissue would allow the association of glomerular, tubular and endothelial damage with SARS-CoV-2 infection.

As previously mentioned, to date no cases of IgA-DIAGN following COVID-19 have been described. GN development could be linked to the cytokine storms that occur in COVID-19. An increase in pro-inflammatory cytokines is a constant in affected patients and it has been postulated that this effect is related to the action of SARS-CoV-2 on the ACE-2 receptor [34]. Moreover, angiotensin II and its receptor (AT1R) axis are mainly associated with vasoconstriction, but the importance of this axis in immunity regulation has also been emphasized for its ability to increase levels of NF-kB and IL-6 [35]. As a consequence, the virus dysregulates the ACE-2 receptor and enters the cell, angiotensin II interacts with AT1R, and can thus trigger a cytokine storm [36]. This burst of cytokines activates B cells which would produce polyclonal IgA and IgG, with CIC formation and glomerular deposition, as has been proposed to occur in IgA-DIAGN [16]. Another study postulated that SARS-CoV-2 spike protein (S) by itself may act as a superantigen [37]. Using structure-based computational models, the authors demonstrate that SARS-CoV-2 S exhibits a high-affinity for binding T cell receptors and interacts closely with the complementarity-determining regions of both α- and β-chain variable domains. Thus, the binding epitope on S harbors a sequence motif unique to SARS-CoV-2 (not present in any other coronavirus), which is highly similar in both sequence and structure to other bacterial superantigens. Further examination revealed that this interaction between viruses and human T cells is strengthened in the context of a recently reported rare mutation (D839Y/N/E) from a European strain of SARS-CoV-2. All these data support that SARS-CoV-2 S may act as a super-antigen, driving the development of multisystem inflammatory syndrome in children as well as cytokine storms in adults affected by COVID-19 [37]. Acting as a superantigen, SARS-CoV-2 S could stimulate the proliferation and massive activation of T cells as well as production of interleukins, TNF and IFN, which would lead to the development of this novel IgA-DIAGN, as occurs with enterotoxin B and C in staphylococcus-related infections [15,18]. In animal models, Rops et al. reported a link between IL-6 levels and glomerular IgA deposits [38]. These finding suggest that IL-6 could play a relevant role in the development of GN-associated infection with dominant IgA. Regarding the endothelial lesions found in our patient, there is increasing evidence pointing to endothelium as a key target in COVID-19, and the resulting endothelial dysfunction as part of the systemic manifestations of the disease [39]. The absence of humps in the biopsy of our patient could be explained by the time lag between the start of the SARS-CoV-2 infection and the renal biopsy (seven weeks). Intriguingly, it has been hypothesized that severe COVID-19 might, partly at least, be an IgA-mediated disease (related to IgA deposit and vasculitis). These findings may explain common organ injuries such as acute pulmonary involvement and kidney damage, among others [40]. Improvement in renal function coinciding with steroid use supports this inflammatory hypothesis.

In summary, this report documents the first case of SARS-CoV-2-associated IgA-DIAGN. SARS-CoV-2 S may act as a superantigen driving the development of multisystem inflammatory syndrome and cytokine storms in patients affected by COVID-19, reaching the glomerulus and leading to the development of this novel IgA-DIAGN. Given the dramatic spread of the COVID-19 pandemic worldwide, further similar cases are expected to appear, highlighting the importance of advancing the understanding of this entity.

## Figures and Tables

**Table 1:** Patient clinical data.

	Baseline (before Hospitalization)	Hospitalization COVID-19	Discharge COVID-19	Hospitalization IgA-CGMN	Hospitalization IgA-CGMN (+6 days)	Discharge IgA-CGMN (+11)	Range
Creatinine (mg/dL)	1.01	1.35	0.83	2.14	3.19	2.35	0.65–1.35
eGFR (ml/min)	66.2	46.6		26.6		23.8	
Urea (mg/dL)		147	36	148	133	117	20–50
Na (mmol/L)	143	136	141	145	139	136	135–145
K+ (mmol/L)	4.6	3.6	4.1	4.4	4.4	4.2	3.5–5.1
Uric Acid (mg/dL)	7.6			4.4		7.1	3.5–7.2
Calcium (mg/dL)	9.9			8.6		8.2	8.1–10.5
Phosphate (mg/dL)				5.3		3.5	2.5–5.0
Magnesium (mg/dL)		2.6		2.4		1.9	1.7–2.6
Ferritin (ng/mL)		1681	664	316			20–300
Albumin (g/dL)	4.2			3		2.8	3.5–5.2
AST (U/L)	17	52	24	22	24	17	1.0–37
ALT (U/L)	20	25	24	11	25	22	1.0–41
Bilirubin (mg/dL)	1.04	0.93		0.77		0.47	0.1–1
LDH (U/L)		863	492	607	569	395	240–480
Procalcitonin (ng/mL)		0.67	0.14	0.1	0.13		0.0–0.5
CRP (mg/L)		220.8	14.7	16.4	14	1.1	0–5
ASLO (UI/mL)						34	<200
WC (109/L)	4.36	9.16	2.96	5.65	4.16	7.01	4.1–11
Lymphocytes	1.03	0.22	0.75	1.23	0.61	1.41	1.5–4.5
Hemoglobin (g/dL)	13.1	9.9	9.9	9.8	9.2	8.9	12.0–16.0
Platelet (x 109)	121	130	96	105	108	158	140–400
IQ (%)		91	97	97	100	92	75–110
D Dimer		824	245	904	4485	458	<250
Immunofixation				Oligoclonal band IgG kappa, IgM-kappa and lambda light chain		Oligoclonal bands IgG kappa, IgM-kappa and lambda light chain	
C3 (mg/dL)				97		83	90–180
C4 (mg/dL)				9		7	10–40
Rheumatoid Factor (UI/mL)				179		170	0–14
URINE							
Nitrites	Neg	Neg		Neg		Neg	Neg
Urobilinogen (mg/dL)		2		0.2		0.2	<2.0 mg/dL
Protein lab stick (mg/dL)	Neg	70		>600		300	<20 mg/dL
RPC				8.38	5.79	5.17	<0.20
pH	5.5	5.5		6		7	5.0–8.0
Density	1.015	1.03		1.03		1.005	1.005
Ketonic bodies	Neg	Neg		Neg		Neg	Neg
Biliary	Neg	Neg		Neg		Neg	Neg
Glucose (mg/dL)	Neg	Neg		Neg		70 mg/dL	<30 mg/dL
RBC	Neg	0–1		>100		>100	Neg
Acanthocytes				20–40%			Neg
WC	Neg	1–5		10–20		1–5	Neg
Cast	Neg	Neg		Granular		Neg	Neg

## Data Availability

Exclude this statement.
